# Monitoring Anti-PEG Antibodies Level upon Repeated Lipid Nanoparticle-Based COVID-19 Vaccine Administration

**DOI:** 10.3390/ijms23168838

**Published:** 2022-08-09

**Authors:** Giuditta Guerrini, Sabrina Gioria, Aisha V. Sauer, Simone Lucchesi, Francesca Montagnani, Gabiria Pastore, Annalisa Ciabattini, Donata Medaglini, Luigi Calzolai

**Affiliations:** 1 European Commission, Joint Research Centre (JRC), Ispra, Italy; 2 Laboratory of Molecular Microbiology and Biotechnology, Department of Medical Biotechnologies, University of Siena, 53100 Siena, Italy; 3 Department of Medical Biotechnologies, University of Siena, 53100 Siena, Italy; 4 Department of Medical Sciences, Infectious and Tropical Diseases Unit, University Hospital of Siena, 53100 Siena, Italy

**Keywords:** lipid-nanoparticle-mRNA (LNP-mRNA)-based vaccine, anti-PEG Ig, COVID-19, anti-Spike Ig, SARS-CoV-2

## Abstract

PEGylated lipids are one of the four constituents of lipid nanoparticle mRNA COVID-19 vaccines. Therefore, various concerns have been raised on the generation of anti-PEG antibodies and their potential role in inducing hypersensitivity reactions following vaccination or in reducing vaccine efficacy due to anti-carrier immunity. Here, we assess the prevalence of anti-PEG antibodies, in a cohort of vaccinated individuals, and give an overview of their time evolution after repeated vaccine administrations. Results indicate that, in our cohort, the presence of PEG in the formulation did not influence the level of anti-Spike antibodies generated upon vaccination and was not related to any reported, serious adverse effects. The time-course analysis of anti-PEG IgG showed no significant booster effect after each dose, whereas for IgM a significant increase in antibody levels was detected after the first and third dose. Data suggest that the presence of PEG in the formulation does not affect safety or efficacy of lipid-nanoparticle-based COVID-19 vaccines.

## 1. Introduction

The approval by the US Food and Drug Administration (FDA) and European Medicines Agency (EMA) of new generation COVID-19 vaccines based on mRNA encapsulated in lipid nanoparticles (LNP-mRNA) developed by BioNTech/Pfizer and Moderna, paved the way for a worldwide use of LNP-mRNA systems in vaccines. This new generation of vaccines represents a powerful and flexible technology which could avoid the risk of anti-vector immunity experienced towards viral vectors (e.g., adenoviral vectors) [1] and require strong characterization process [2]. The LNP carriers used in both vaccine formulations contain PEGylated lipids (ALC-0159 or PEG2000 DMG in the case of BioNTech/Pfizer or Moderna, respectively), in addition to three other non-PEGylated lipid components [3]. These PEGylated lipids consist of a long lipid tail and polyethylene glycol (PEG), a synthetic polymer made of repeated units of ethylene glycol.

PEG usually prevents non-specific protein adsorption; it increases drug half-life in plasma (avoiding macrophage recognition) and in general confers “stealth” properties to the formulation [4]. Moreover, in the case of nanopharmaceuticals (such as the liposomal anticancer drug Doxil^®^, Johnson & Johnson, New Brunswick, NJ, USA), PEG coatings contribute to the colloidal stability of the nanoparticles and prevents aggregation, improving the safety profile and facilitating cargo delivery. PEG is considered biologically inert [5], and due to its physico-chemical characteristics and low toxicity, it is currently contained in more than one thousand FDA-approved medications, from tablets to topical gels, laxatives and parenteral steroids [6]. To date, fourteen PEGylated biopharmaceuticals have been approved in Europe and the United States, across multiple indications [4,7,8]. As more PEG-containing drugs become approved for human use [9], there have been increasing concerns that the generation of anti-PEG antibodies could potentially lead to hypersensitivity reactions [10,11], especially following repeated treatment with PEG-containing products or PEGylated drugs, that could affect vaccine efficacy.

Anti-PEG antibodies can be detected in the general population, although different studies reported a wide range of prevalence (from 24% to 65–76%) [12,13,14]. In rare occasions, severe effects have been reported in response to continued or repeated somministrations [14], mainly in the case of PEGylated nano-biopharmaceuticals [15]. The generation of anti-PEG antibodies could also contribute to a reduced therapeutic efficacy of biological drugs [15]. Considering that multiple administrations of LNP-mRNA vaccines are needed to induce a protective immune response, concerns about possible hypersensitivity reactions to PEG were also raised early on in the COVID-19 pandemic [10,16], and cases of allergic reactions have been reported in the literature [17,18,19]. A recent study correlated an episode of anaphylactic reaction after the BioNTech/Pfizer vaccine in a woman with high anti-PEG antibody levels, determined by a skin prick test. However, the study also showed that three other subjects, experiencing a similar systemic adverse reaction, did not present pre-existing anti-PEG antibodies [20].

The two approved LNP-mRNA vaccines have shown excellent safety and efficacy in phase-III clinical trials and, since the initiation of the massive vaccination campaigns against SARS-CoV-2, only rare cases of immediate allergic reactions have been reported (11.1 and 2.5 cases per million of vaccine doses for the BioNTech/Pfizer and Moderna vaccines, respectively) [21]. As the perception of vaccination-related risks has been greatly amplified by the extensive reporting echo in newspapers, television, and social media, this has contributed to the dilution of encouraging messages published by the scientific community on the safety and efficacy of these vaccines [22,23].

Population studies on the prevalence of anti-PEG antibodies and their evolution following repeated administration of LNP-mRNA vaccines could support or reinstate trust in the safety of these vaccines in vaccine-hesitant populations. Such studies could also contribute to determine a cut-off value for diagnostic purposes, to reliably identify any at-risk individual that could be susceptible to the extremely rare events of anaphylaxis in response to RNA COVID-19 vaccines [24]. Finally, such data could also help establishing a possible correlation between the presence of anti-PEG antibodies and vaccine efficacy.

Here, we assess the presence of anti-PEG antibodies, IgG and IgM, in a cohort of vaccinated individuals, and give an overview of their time evolution after repeated vaccine administration, evaluating any sign of possible reduction in vaccine immunogenicity. Furthermore, potential differences between younger and older populations have been considered.

## 2. Results

**Figure 1:**
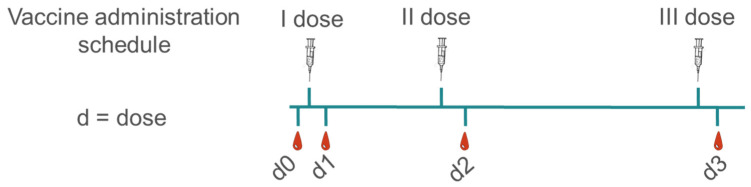
Vaccination schedule and blood sample collection: serum samples were collected before vaccination (d0), and seven days after each dose of vaccine (d1, d2 and d3). Vaccine schedule was I and II dose 21 days apart, and III dose 6 months following the II dose. All volunteers received BioNTech/Pfizer as t I and II dose, whereas for the III dose either BioNTech/Pfizer or a half-dose of Moderna were administered.

Samples were collected from sixty-nine healthy volunteers (HCWs), 79.8% female and 20.2% male, aged from 25 to 80 years old (61.66% below 50y, 38.33% above 50y old), who received three doses of LNP-mRNA vaccines, following the vaccination schedule reported in Figure 1, and were analysed for serum level of anti-PEG IgG and anti-PEG IgM using commercially available ELISA kits. Volunteers received the first vaccination cycle (first and second doses) with BNT162b2 (BioNTech/Pfizer, Comirnaty) vaccine and were boosted with BNT162b2 (34.32%) or with mRNA-1273 (Moderna; 65.68%) as the third dose.

IgM are mainly produced in the early phase after the first exposure to a particular antigen and are rapidly replaced (mean seroconversion 10 days) by IgG, which represents a later-stage response and ensures long-term humoral response [25]. In our study, anti-PEG IgG and anti-PEG IgM were measured as baseline before vaccination (d0) and 7 days post first, second and third vaccine dose (d1, d2 and d3, respectively), as reported in Figure 1.

**Figure 2:**
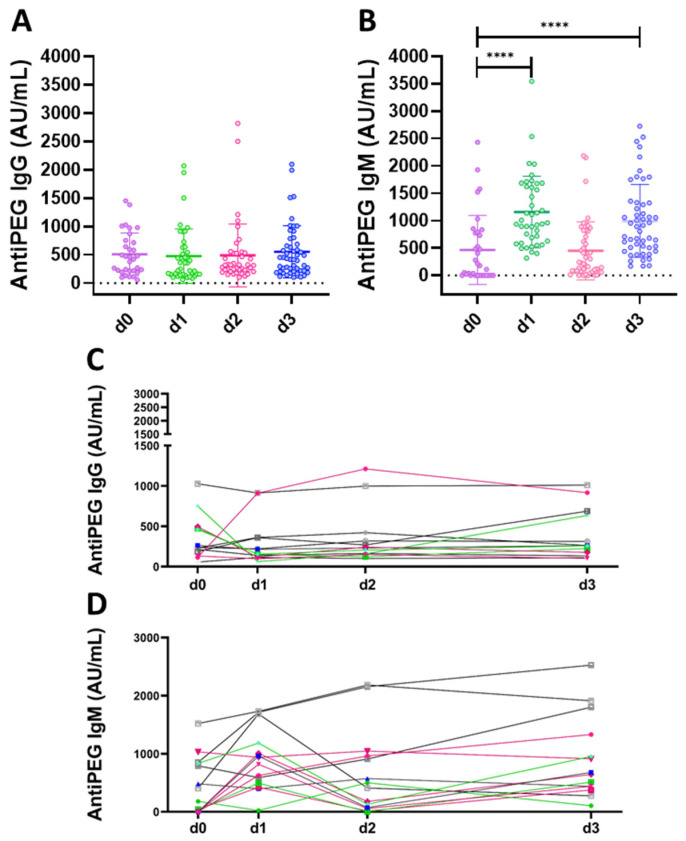
Anti-PEG antibodies following lipid nanoparticles-mRNA COVID-19 vaccination (BioNTech/Pfizer or Moderna). Anti-PEG IgG (**A**) and anti-PEG IgM (**B**) measured in heathy donors before vaccination and seven days after each vaccine dose (d0, d1, d2 and d3, respectively). Longitudinal analysis of anti-PEG IgG (**C**) and anti-PEG IgM (**D**), different colours represent different healthy donors. Kruskal–Wallis test, followed by Dunn’s post-test for multiple comparisons, was used for assessing statistical differences between groups (**** *p* ≤ 0.0001).

Figure 2 shows the serum levels of anti-PEG IgG (Figure 2A) and anti-PEG IgM (Figure 2B) antibodies, seven days after each vaccine dose, compared with the basal level before vaccination. Results indicate that in our cohort, anti-PEG IgG are already present before the first vaccine administration (d0 mean value 509.07 AU/mL), with a large person-to-person variability: 37.5% of the cohort show very low levels (below 250 AU/mL), 50% have intermediate values (between 250 and 1000 AU/mL) and 12.5% high levels (above 1000 AU/mL) of anti-PEG IgG. Seven days after the first, second and third vaccine dose (d1, d2 and d3, respectively), the average levels of anti-PEG IgG remained stable, with values of 477.78 AU/mL, 539.55 AU/mL and 547.72 AU/mL, respectively, showing no significant increase in antibody titre. To note that, at individual level, 15.38% of individuals already show high antibody levels before vaccination, close to and above 1000 AU/mL, value that is considered as potentially worrying threshold for anaphylactic reaction according to previous studies [24]. In our cohort, the first vaccine shot caused an increase in approximately two-folds of anti-PEG IgG levels (compared with d0) in 15.38% of subjects, of which nobody reached values above 1000 AU/mL. The second and third shot doubled anti-PEG IgG levels in 15.8% and 23.07% of individuals, respectively (with respect to d0), of which 7.6% were above 1000 AU/mL in both cases (Figure 2C).

In the case of anti-PEG IgM, before vaccination, 43.7% of donors show almost no detectable antibodies (<100 AU/mL), 40.6% have intermediate values and 15.6% show high values (above 1000 AU/mL). d1 caused an increase in the average value of anti-PEG IgM from 472 AU/mL measured at d0 to 1162 AU/mL, indicating a substantial production of IgM antibodies, most likely due to the presence of PEGylated lipids in the vaccine formulation. In particular, 47.6% of the samples had values above 1000 AU/mL. d2 showed a decrease in the average anti-PEG IgM value to 482 AU/mL, which increased again after the third dose to 995 AU/mL (with 43.1% above 1000 AU/mL).

At individual level, after the first vaccine shot, 69.23% doubled the antibody titer and 38.46% of individuals reached values above the 1000 AU/mL (of which, 80% had already high antibody level at d0). The second dose doubled the antibody levels in 46.15% of individuals compared with d0. Overall, 23.07% of individuals reached 1000 AU/mL at d2 and all these subjects already showed high antibody levels at d0. The third dose doubled the antibody levels in 61.53% of individuals compared with d0, and 23% showed antibody levels upon 1000 AU/mL, of which 66.6% already had high antibody levels at d0 (Figure 2D).

Time course analysis of anti-PEG IgG and IgM values are represented in Figure S1, showing fluctuating IgM levels in most of the volunteers, after vaccine shots d1 and d3, with respect to d2 (which decreases towards levels detected at d0). Conversely most of the volunteers showed no significant increase in anti-PEG IgG compared with the basal level (d0), and did not show a booster effect after subsequent vaccine shots, independently from the age.

**Figure 3:**
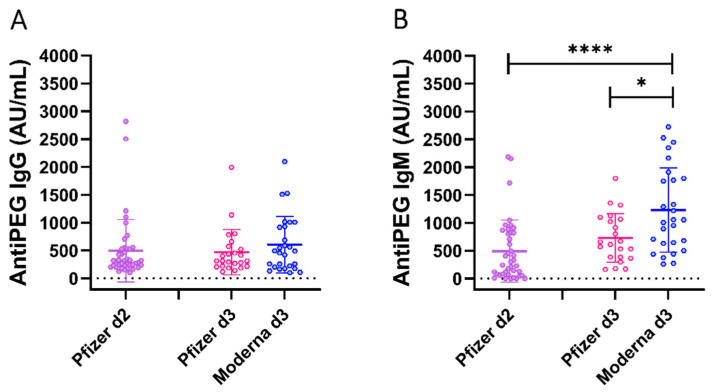
Comparison of anti-PEG IgG and IgM in subjects boosted with BioNTech/Pfizer or Moderna formulation. Anti-PEG IgG (**A**) and anti-PEG IgM (**B**) measured in heathy donors. All the volunteers received the BioNTech/Pfizer vaccine as the first and second doses, and then were boosted with Pfizer-BioNTech (Pfizer d3, 34.3%) or Moderna (Moderna d3, 65.7%) for the third dose. Kruskal–Wallis test, followed by Dunn’s post-test for multiple comparisons, was used for assessing statistical differences between groups (* *p* ≤ 0.05; **** *p* ≤ 0.0001).

The present cohort has been vaccinated with the BioNTech/Pfizer vaccine formulation for the first and second vaccination, but with either BioNTech/Pfizer or Moderna as the third dose. Regarding these different vaccine formulations, we observe no significant increase in the level of anti-PEG IgG between d2 and d3, whereas the level of anti-PEG IgM after d3 was statistically significant higher for those who received the Moderna vaccine compared with the BioNTech/Pfizer vaccine formulation (Figure 3).

**Figure 4:**
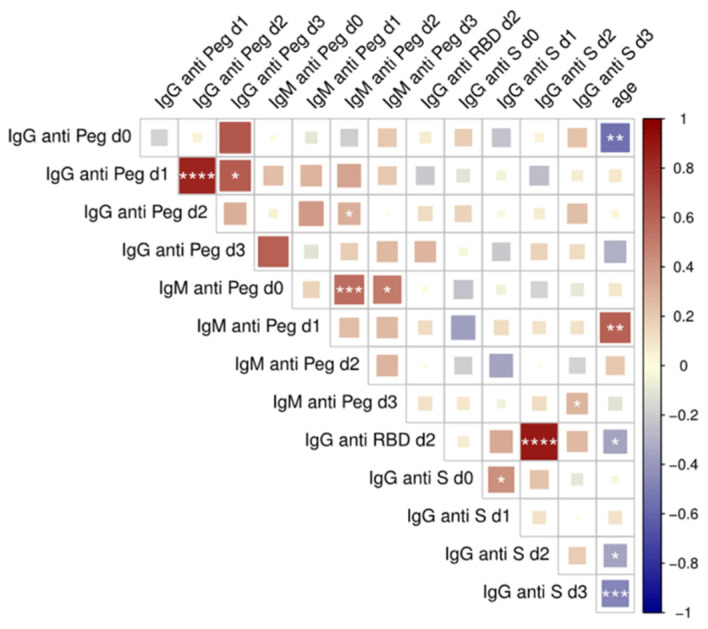
Correlation analysis between anti-PEG, anti-Spike, anti-RBD antibodies and age. Multiple correlations were visualized as matrix with the Spearman’s correlation coefficient values reported. Correlation coefficients were reported with a color scale between red (direct correlation) and blue (inverse correlation) Color intensity and square size are proportional to the absolute value of Spearman correlation coefficients. Statistically significant correlations were labeled as: * *p* ≤ 0.05; ** *p* ≤ 0.01; *** *p* ≤ 0.001; **** *p* ≤ 0.0001.

To assess whether the substantial increase in anti-PEG IgM observed after the first vaccination influences vaccine efficacy, we correlated it with the level of anti-Spike and anti-Receptor Binding Domain IgG (RBD, the portion of the spike protein which binds to the host receptor ACE-2) measured in the same subjects, at the same time points as reported in a previous study [25]. The working hypothesis is that an elevated level of anti-PEG antibodies could reduce the vaccine immunogenicity, leading to a lower amount of anti-Spike (or anti-RBD) antibodies. Figure 4 reports the correlation coefficients among the levels of anti-PEG IgG, IgM and anti-Spike IgG and anti-RBD IgG. No correlation was found between anti-PEG antibodies and anti-Spike/RBD antibodies [25], whereas anti-PEG IgM at d1 positively correlated with age, probably due to higher sensitization rates to repeated exposure to PEG-containing products, such as cosmetics, over time.

Other statistically significant correlations were observed between anti-PEG IgG levels after d1 and d2 and those between the anti-Spike IgG and anti-RBD IgG (as expected). Similar analysis performed on the subgroup of individuals with high levels of anti-PEG antibodies also did not reveal any statistically relevant correlation with the levels of anti-Spike or anti-RBD antibodies.

## 3. Discussion

Both approved LNP-mRNA COVID-19 vaccines contain PEGylated lipids in their formulations. It has been shown that prolonged exposure to PEG could trigger hypersensitivity up to rare cases of anaphylactic reaction, caused by the production of anti-PEG-specific antibodies. Therefore, various concerns had been raised related to the safety use of these vaccines [26,27]. As PEG is present in several medications as well as in cosmetics or other products for human use, we first measured the anti-PEG Ig levels at baseline (d0). Data at d0 confirm the presence of anti-PEG IgG antibodies in the entire cohort, although a large person-to-person variability was observed. These results are in line with published works that indicate a large prevalence of anti-PEG IgG in the general population [12,14].

Furthermore, 12.5% of the cohort already presented levels above 1000 AU/mL at d0, which could be considered a worrying threshold [24] for anaphylactic reactions.

Yu et al. analysed and compared Anti-PEG IgG and IgM in adults who received two doses of BNT162b2 vaccine or two doses of mRNA-1273. They concluded that the Moderna vaccine significantly boosted both anti-PEG IgG and IgM, compared with BNT162b2 vaccination, which showed only modest boosting effect [28]. In our study, time-course analysis of anti-PEG IgG showed no significant booster effect after each dose, whereas for IgM a significant increase in antibody levels at d1 and d3 was detected. Of note, the time points analysed were different between the two studies, and this could possibly contribute to the discrepancy in the results. The increment of anti-PEG IgM that we observed was expected at d1 since IgMs are involved in early immunological response to infection. Over time, IgM+ B cells undergo isotype switching and are replaced by cells secreting IgG; however, in this study, an increase in anti-PEG IgM was observed also after d3. According to them, Moderna vaccination elicited a stronger boost effect even in our cohort, although only a half-dose was administered as the third dose.

It is important to note the different vaccine administered in our cohort, according to the availability during the emergency state of the pandemic. The high IgM level registered with d3 seems to be linked to the different vaccines administered (BioNTech/Pfizer as d1, either BioNTech/Pfizer or Moderna as d3) rather than to the prolonged time which occurred between d2 and d3. Indeed, the increment was significantly higher in volunteers who received the Moderna vaccine as the third dose with respect to the ones who received BioNTech/Pfizer.

The reported lipid formulations of the BioNTech/Pfizer and Moderna vaccine, although quite similar, use slightly different PEGylated lipids (PEG2000-ALC-0159 and PEG2000-DMG, respectively) [29]. In addition, they contain different amount of mRNA: 100 ug/dose for Moderna and 30 ug in the case of BioNTech/Pfizer. In our cohort, the heterologous vaccination schedule foresees a half-dose of the Moderna vaccine as d3. Given that the ratio of lipid to mRNA is generally kept constant, it can be estimated that, even with the half-dose of the Moderna vaccine administered at d3, individuals receiving the Moderna vaccine would be exposed to a higher amount of PEG compared with those receiving the BioNTech/Pfizer vaccine. This could explain the increment of anti-PEG IgM in subjects who received the Moderna vaccination at the d3.

Despite the increased levels of anti-PEG IgM, none of the tested individuals (according to a study survey) reported serious adverse effects upon vaccination. It also needs to be highlighted that the more severe immune reactions to PEGylated nano-pharmaceuticals occurred following intravenous injection, whereas vaccines are administered intramuscularly, thus probably contributing to a better safety profile. Results obtained also revealed no significant difference in anti-PEG IgG levels based on the administered vaccine.

These results are in-line with recent, non-peer reviewed results in the bioarchive that indicates substantially higher anti-PEG antibodies after vaccination with the Moderna vaccine, compared with the BioNTech/Pfizer ones.

Time course analysis of anti-PEG IgG and anti-PEG IgM confirmed that no booster effect was observed after repeated vaccinations, and this was confirmed to not be influenced by age or gender. Our findings support recent evidence that the role of PEG in the occurrence of anaphylactic reactions to COVID-19 vaccine is marginally low or extremely rare [19].

In respect of concerns that the presence of anti-PEG antibodies could reduce the immunogenicity of the vaccine, no statistically relevant correlation was found between the levels of anti-PEG and anti-Spike antibodies after each vaccination. This suggests that the production of anti-Spike (and anti-RBD) antibodies is not influenced by the generation of anti-PEG IgM against PEGylated lipids in the vaccine formulation. This was confirmed also in the case of the heterologous vaccination schedule, where two BioNTech/Pfizer doses were followed by a Moderna dose [25].

Anti-PEG Ig values were measured by ELISA, using commercially available kits, which can be easily implemented following the manufacturer’s instructions. Such assays can support measuring anti-PEG levels in patients suspected to be at high risk for an adverse reaction; for example, patients undergoing therapies with PEG-containing drugs [9]. Unfortunately, anti-PEG antibodies are routinely not measured in clinic and, due to the lack of a reference standard, they would also be of help when testing the specificity of the antibody response. It still remains difficult to normalise data obtained when using kits from different producers.

In this context, inter-laboratory comparisons and the development of reference standards for anti-PEG antibodies could improve the comparability of measurement results and lead to the establishment of a critical anti-PEG antibody threshold.

Based on previous studies, we considered a response of 1000 AU/mL and above as a possible warning level of anti-PEG antibodies. However, in the examined cohort, even in individuals with values above 1000 AU/mL at d0, no severe adverse reactions were reported. Setting a standardized threshold for possible risk of anaphylactic reaction could contribute to determining a cut-off value for diagnostic purposes, and to reliably identify any at-risk individual that could be susceptible to the extremely rare event of anaphylaxis to LNP-mRNA COVID-19 vaccine.

Our results support or reinstate trust in the safety of LNP-mRNA vaccines in vaccine-hesitant population. The main prerequisites for vaccines are safety and efficacy: here, we demonstrate that, in the cohort tested, the presence of PEG in the formulation did not compromise the safety of BioNTech/Pfizer and Moderna COVID-19 vaccines nor influenced its immunogenicity.

## 4. Material and Methods

### 4.1. Plasma Samples

Blood samples were obtained from sixty-nine volunteers (HCWs) who received three doses of LNP-mRNA vaccines (BNT162b2/Comirnaty, BioNTech (Mainz, Germany)/Pfizer (New York, USA)) or mRNA-1273 (Moderna)) according to the approved vaccination scheme (second dose 21 days after the first and third dose 6 months from the second). The entire cohort received BioNTech/Pfizer as first and second doses, whereas they were boosted with BioNTech/Pfizer or Moderna. In the case of the Moderna vaccine used as a booster, only a half-dose was applied.

Samples were collected in green cap-BD Vacutainer^®^ (Franklin Lakes, NJ, USA) blood collection tubes at time 0 (before vaccination, d0) and then seven days after the first, second and third dose (d1, d2 and d3, respectively). Plasma fractions were stratified with Lymphoprep™ (Alere Technologies, Waltham, MA, USA) and stored at −80 °C. Exclusion criteria included pregnancy, previous documented SARS-CoV-2 infection, and immunocompromising comorbidities (congenital, acquired, or drug-related). Study participants were recruited at the Infectious and Tropical Diseases Unit, Azienda Ospedaliera Universitaria Senese (Siena, Italy) upon written informed consent. The study was performed in compliance with the Human Tissue Act (2004), the Human Tissue Authority Code of Practice 1 (April 2005) and the protocol was approved by the local Ethical Committee for Clinical experimentation of Regione Toscana Area Vasta Sud Est (protocol code 18869 IMMUNO_COV, v1.0 approved on 21 December 2020). Clinical data collection, self-reported side effects survey and sample management were carried out using the software REDCap (Research Electronic Data Capture, Vanderbilt University).

### 4.2. Enzyme-Linked Immunosorbent Assay

Anti-PEG IgG and anti-PEG IgM antibodies were detected with Human Anti-PEG IgG and IgM ELISA kit (PEGG-20 and PEGM-20, respectively; Gentaur, Kampenhout, Belgium). The kits were used according to the manufacturer’s instructions. All samples were run in duplicated and diluted 1:20. Optical density was measured for both assays at 450 nm and 620 nm (the latter for background subtraction) using a Multiskan Microplate Spectrophotometer (Thermo Scientific, Waltham, MA, USA). The antibody levels were calculated using the calibration curve built with the standards according to the protocol provided by the manufacturer. Results are reported as arbitrary units per unit volume (AU/mL).

Anti-spike and RBD IgG response was tested on Maxisorp microtiter plates (Nunc, Denmark) coated with recombinant SARS-CoV-2 Spike S1 + S2 ECD or Spike-RBD (all from Sino Biological, Chesterbrook, PA, USA), as previously reported [30].

### 4.3. Statistics

The Kruskal–Wallis test, followed by Dunn’s post-test for multiple comparisons, was used for assessing statistical differences between groups with asterisks indicating different levels of significance (* *p* ≤ 0.05; ** *p* ≤ 0.01; *** *p* ≤ 0.001; **** *p* ≤ 0.0001). Multiple correlations analysis between anti-PEG, anti-Spike and anti-RBD antibodies were performed with the nonparametric Spearman rank correlation. The Mann–Whitney U test was used to assess statistical differences in anti-Spike and anti-RBD antibody levels in individuals with high level of IgM or IgG anti-PEG (>1000 AU/mL, see results and discussion). Fisher’s exact test was used to test odds ratio between high levels of IgM anti PEG at d1 and IgG anti-Spike at d2.

## Data Availability

All data related to the study are published as supplementary material.

## Supplementary Materials

The supporting information can be downloaded at: https://www.mdpi.com/article/10.3390/ijms23168838/s1.
