# Supplementary material for: Monitoring Anti-PEG Antibodies Level upon Repeated Lipid Nanoparticle-Based COVID-19 Vaccine Administration
Source: Int J Mol Sci. 2022 Aug 9;23(16):8838. doi: 10.3390/ijms23168838 (PMC9408675; doi:10.3390/ijms23168838)
Supplement: Supplementary file 1 [file ijms-23-08838-s001.zip › ijms-1829939-supplementary/supplementary material/Supplementary material-Figure S1.pdf]

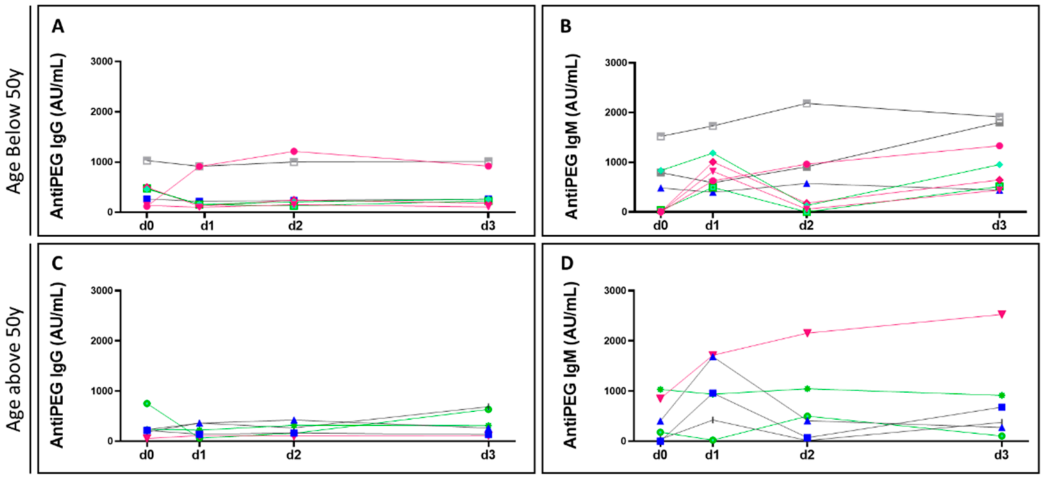

Figure S1: Longitudinal analysis of anti-PEG IgG (A, C) and anti-PEG IgM (B, D) in subjects < 50 years old (A,B) and > 50 years old (C,D), different colours represent different healthy donors.
